# The monomeric C-reactive protein level is associated with the increase in carotid plaque number in patients with subclinical carotid atherosclerosis

**DOI:** 10.3389/fcvm.2022.968267

**Published:** 2022-07-22

**Authors:** Ivan Melnikov, Sergey Kozlov, Olga Pogorelova, Maria Tripoten, Leyla Khamchieva, Olga Saburova, Yuliya Avtaeva, Maria Zvereva, Evgeny Matroze, Tatiana Kuznetsova, Lyudmila Prokofieva, Tatiana Balakhonova, Zufar Gabbasov

**Affiliations:** ^1^Laboratory of Cell Hemostasis, National Medical Research Centre of Cardiology named after academician E.I. Chazov of the Ministry of Health of the Russian Federation, Moscow, Russia; ^2^Laboratory of Gas Exchange, Biomechanics and Barophysiology, State Scientific Center of the Russian Federation – The Institute of Biomedical Problems of the Russian Academy of Sciences, Moscow, Russia; ^3^Laboratory of Problems of Atherosclerosis, National Medical Research Centre of Cardiology named after academician E.I. Chazov of the Ministry of Health of the Russian Federation, Moscow, Russia; ^4^Department of Ultrasound Diagnostics, National Medical Research Centre of Cardiology named after academician E.I. Chazov of the Ministry of Health of the Russian Federation, Moscow, Russia; ^5^Department of Innovative Pharmacy, Medical Devices and Biotechnology, Moscow Institute of Physics and Technology, Moscow, Russia; ^6^Laboratory of Neurohormonal Regulation of Cardiovascular Diseases, National Medical Research Centre of Cardiology named after academician E.I. Chazov of the Ministry of Health of the Russian Federation, Moscow, Russia; ^7^Department of Cardiology, Functional and Ultrasound Diagnostics, Sechenov University, Moscow, Russia

**Keywords:** monomeric C-reactive protein (mCRP), hsCRP, inflammatory biomarkers, residual inflammatory risk, carotid atherosclerosis, plaque number, plaque height

## Abstract

The high-sensitivity C-reactive protein (hsCRP) assay measures the level of the pentameric form of CRP in blood. Currently, there are no available assays measuring the level of the monomeric form of CRP (mCRP), produced at sites of local inflammation. We developed an assay measuring the mCRP level in blood plasma with functional beads for flow cytometry. The assay was used to measure the mCRP level in 80 middle-aged individuals with initially moderate cardiovascular SCORE risk. By the time of the mCRP measurement, the patients have been followed up for subclinical carotid atherosclerosis progression for 7 years. Ultrasound markers of subclinical atherosclerosis, which included plaque number (PN) and total plaque height (PH), were measured at baseline and at the 7th-year follow-up survey. Inflammatory biomarkers, including mCRP, hsCRP, inteleukin-6 (IL-6) and von Willebrand factor (VWF) level, were measured at the 7th-year follow-up survey. The median level of mCRP was 5.2 (3.3; 7.1) μg/L, hsCRP 1.05 (0.7; 2.1) mg/L, IL-6 0.0 (0.0; 2.8) pg/mL, VWF 106 (77; 151) IU/dL. In the patients with the mCRP level below median vs. the patients with the median mCRP level or higher, change from baseline in PN was 0.0 (0.0; 1.0) vs. 1.0 (1.0; 2.0) and PH 0.22 (−0.24; 1.91) mm vs. 1.97 (1.14; 3.14) mm, respectively (*p* < 0.05). The adjusted odds ratio for the formation of new carotid atherosclerotic plaques was 4.7 (95% CI 1.7; 13.2) for the patients with the median mCRP level or higher. The higher mCRP level is associated with the more pronounced increase in PN and PH in patients with normal level of traditional inflammatory biomarkers and initially moderate cardiovascular SCORE risk.

## Introduction

The complications of atherosclerosis are among the leading causes of disability and mortality worldwide. One of the most advocated measures to prevent or delay atherosclerosis development is the reduction of the low-density lipoprotein cholesterol (LDL-C) level in blood (1). Yet, even extreme reduction in the LDL-C level cannot prevent major adverse cardiovascular events (MACE) (2). A crucial contributor to cardiovascular risk that remains in spite of aggressive lipid-lowering and control of other modifiable risk factors is low-grade inflammation in atherosclerotic plaques (3). Currently, the level of the main inflammatory biomarker C-reactive protein measured by the high-sensitivity assay (hsCRP) ≥ 2.0 mg/L is considered as a factor increasing estimated cardiovascular risk (4). Large randomized controlled clinical trials CANTOS, COLCOT, LoDoCo2 demonstrated that the reduction of the hsCRP level below 2.0 mg/L with anti-inflammatory agents decreased the MACE frequency by 23–29% (5). The hsCRP assays detect the plasma concentration of the pentameric form of CRP (pCRP), which is synthesized in hepatocytes under stimulation by proinflammatory cytokines, predominantly interleukin-6 (IL-6) (3). At sites of local inflammation pCRP binds to its specific ligand, lysophosphatidylcholine, on membranes of damaged or apoptotic cells and microparticles, as well as oxidized LDL, and undergoes dissociation to the monomeric form of CRP (mCRP) (6, 7). Dissociation opens up terminal octapeptide (Phe-Thr-Lys-Pro-Gly-Leu-Trp-Pro) on the C-terminal end of monomeric subunits, giving mCRP its antigenic specificity (6, 8, 9). After dissociation mCRP remains predominantly bound to cell membranes (10, 11). mCRP was found on circulating microparticles in blood of patients with acute myocardial infarction (12) and peripheral artery disease (13). The mCRP deposits were found in carotid atherosclerotic plaque tissue, but not in the intact vessel wall (14, 15). The mCRP deposits were localized to the necrotic core, neovessels, areas of macrophage, T-cells and smooth muscle cells accumulation in atherosclerotic plaques (14, 15). A range of proinflammatory actions of mCRP was shown *in vitro*. It stimulated monocyte and lymphocyte recruitment and synthesis of proinflammatory cytokines IL-6, IL-8, macrophage and T-cell polarization into proinflammatory phenotype, promoted angiogenesis (10, 16).

However, a feasible commercially available assay measuring the mCRP level in blood plasma or serum is lacking. To date, there are four published papers that describe measuring the mCRP level in blood plasma or serum (17–20). In all these studies the assays were performed with enzyme immunoassay (ELISA) technique. In 2015 Wang et al. produced a monoclonal antibody (mAb) to mCRP and developed an assay for measuring the mCRP level in blood plasma (17). The assay was used to measure the mCRP level in patients with acute myocardial infarction, unstable and stable angina pectoris (17). In 2018 Zhang et al. used the mAb clone 8C8 to measure the mCRP level in blood plasma of patients with autoimmune skin diseases (eczema, psoriasis and urticaria) and healthy controls (18). In 2020 Williams et al. used the mAb clone 8C8 to measure the mCRP level in serum of patients with acute inflammation and the hsCRP level more than 100 mg/L (19). In 2021 Munuswamy et al. used an aptamer-based mCRP competition ELISA to measure the mCRP level in patients with chronic obstructive pulmonary disease (COPD) (20). Previously, the mAb clone 8C8 was used to detect the mCRP deposits in atherosclerotic plaque samples by Jabs et al. (21) and Krupinski et al. (15). Schwedler et al. demonstrated that the mAb clone 8C8 was highly specific to mCRP (22). So far, there were no studies evaluating the association of the mCRP level with ultrasound markers of carotid atherosclerosis.

## Materials and methods

### Study design

The level of mCRP and other inflammatory biomarkers was measured in blood plasma from patients enrolled in the study of subclinical carotid atherosclerosis progression that has been going in the National Medical Research Center of Cardiology, Moscow, Russia, since 2012. This study was initially designed to investigate the benefit of different statin doses in middle-aged persons with moderate cardiovascular SCORE risk, mildly elevated LDL-C level and subclinical non-stenotic carotid atherosclerotic plaques.

Patients of both sexes 40–65 years old with moderate cardiovascular SCORE risk, the LDL-C level 2.7–4.8 mmol/L and subclinical low-grade carotid stenoses narrowing the arterial lumen <50% were eligible for the enrollment.

The study excluded from the enrollment patients with coronary artery disease (CAD), transient ischemic attacks and history of cerebrovascular accidents, symptomatic atherosclerosis of peripheral arteries, atherosclerosis of the carotid and peripheral arteries narrowing the arterial lumen ≥ 50%, aortic aneurysm, diabetes mellitus types 1 and 2, familial hypercholesterolemia, arterial hypertension, chronic kidney disease (GFR <60 ml/min/1.73 m^2^ or serum creatinine > 150 μmol/L), the LDL-C level ≥ 4.9 mmol/L and ≤ 2.6 mmol/L, the triglyceride (TG) level > 4.5 mmol/L, the three-fold or higher increase in the level of aspartate aminotransferase (AST) and/or alanine aminotransferase (ALT) above the upper limit of normal, cardiovascular SCORE risk ≥ 5%, chronic inflammatory diseases (including autoimmune disorders), malignant neoplasms, allergic reactions, lipid-lowering therapy in the previous 12 months, contraindications to statin administration.

Patients underwent an interview, physical examination, full blood count and biochemical blood test, the lipid panel test, electrocardiogram, echocardiogram and carotid ultrasonography. Patients meeting the inclusion criteria were offered to participate in the study. Recruited patients were prescribed atorvastatin with the individual dose adjustment to achieve the target LDL-C level <2.6 mmol/L according to ESC/EAS Guidelines for the Management of Dyslipidaemias that were applicable at the time of enrollment (23). The target LDL-C level was shifted to <1.8 mmol/L in 2019 according to the updated ESC/EAS Guidelines for the Management of Dyslipidaemias (24). The follow-up surveys included annual lipid panel tests and consultations of the same supervising physician. At the 7th-year follow-up survey, all the patients underwent carotid ultrasonography. Assessment of the level of inflammatory biomarkers (mCRP, hsCRP, IL-6) and von Willebrand factor (VWF) in blood plasma was also performed at the 7th-year follow-up survey.

Study enrollment began in 2012 and was completed in 2013. Of 379 consecutively screened individuals, 112 were included in the study. During the first year of the follow-up, 32 participants withdrew from the study. The resulting study population comprised 80 patients, including 47 men (59%) and 33 women (41%). All the patients completed the 7-year follow-up period.

### Carotid ultrasonography

All scanning and reading procedures were identical at baseline and at the 7th-year follow-up survey. All measurements were performed with iU-22 (Phillips, the Netherlands) ultrasound system equipped with a 3–9 MHz linear-array transducer by the same operator at baseline and follow-up. Measurements were performed in B-mode imaging, Color Doppler imaging, Power doppler imaging and Pulsed Wave Doppler mode. The common carotid artery (CCA), the internal carotid artery (ICA) and the carotid artery bifurcation on both sides were scanned for atherosclerotic plaques (6 segments in total) in the anterior, lateral and posterior planes. According to the Mannheim Carotid Intima-Media and Plaque Consensus, a carotid atherosclerotic plaque was defined as a focal structure protruding into the arterial lumen at least 0.5 mm, or at least 50% compared to the adjacent intima-media thickness (IMT), or as a focal intima-media thickening > 1.5 mm (25). Plaque number (the total number of atherosclerotic plaques) and total plaque height (the sum of all plaque heights) were calculated in all 6 examined segments (26). IMT was defined as the distance between the lumen-intima and the media-adventitia interfaces. CCA-IMT was measured along the posterior wall of the distal segments of both CCAs at a distance 1 cm proximal to the bifurcation. Three measurements were performed with the anterior and lateral scanning approach on each side. The highest of 6 mean values calculated from 3 consecutive anterior and 3 consecutive lateral measurements was taken as right or left CCA-IMT. The mean CCA-IMT was calculated as half-sum of right and left CCA-IMT.

### The assay measuring the mCRP level in blood plasma

The assay is based on the Cytometric Bead Array (CBA) kit (BD Biosciences, USA), containing fluorescent functional beads. This kit allows conjugating the beads to an antibody against a target protein. Covalent binding of an antibody to the beads is performed with Sulfo-SMCC (Sigma-Aldrich, USA) and dithiothreitol (Thermo Fisher, USA) according to the manufacturer's protocol. When the complex of antibody and beads is added into a sample of blood plasma, the antibody binds a ligand protein. Then, a fluorochrome-labeled second-layer developing antibody against the same protein is added into the sample. This antibody binds the target protein in the complex with the beads and primary antibody. The intensity of the fluorescence from the second-layer antibody allows quantifying the level of the target protein in the sample by a flow cytometer. The fluorescent spectra of the second-layer antibody and the beads must be different. The CBA beads series A5, C4, and E5 that have different fluorescence intensity in the APC-Cy7 channel were used in this study. The different fluorescence intensity of the beads allows distinguishing between the different series of the beads on a flow cytometry histogram. The A5 beads were conjugated to the anti-pCRP/mCRP mAb clone MOH328 (ImTek, Russia); the C4 beads were conjugated to the anti-mCRP mAb clone 8C8 (Sigma-Aldrich, USA); the E5 beads were conjugated to the anti-pCRP mAb clone MOH372 (ImTek, Russia). The beads conjugated to the anti-pCRP antibodies were used to rule out cross-reactivity of the mAb clone 8C8 to pCRP. As a second-layer developing antibody a FITC-labeled polyclonal goat-antihuman antibody to CRP GAHCRP-FITC (ImTek, Russia) was used. To confirm specificity of the mAbs and construct a calibration curve, pCRP (Sigma-Aldrich, USA) and the recombinant mCRP (a gift by dr. L. Potempa, Roosevelt University, USA) solutions were used. Prior to flow cytometry analysis the beads-antibody conjugates were incubated with studied samples at room temperature for 1 h. All measurements were performed on the FACS CantoII (BD Biosciences, USA) flow cytometer.

### Blood samples collection and analysis

Blood samples were collected from the cubital vein into the S-Monovette 3.8% sodium citrate vials (Sarstedt, Germany) after 12 h of fasting. Platelet poor plasma (PPP) was prepared by centrifugation for 20 min at 2,000 g and stored at −70°C. Before measurements plasma was thawed in an ultrasonic bath at 37°C. The level of hsCRP, IL-6, VWF and the lipid panel were measured at the laboratory of clinical biochemistry of the National Medical Research Centre of Cardiology named after academician E.I. Chazov of the Ministry of Health of the Russian Federation, Moscow, Russia.

### Statistical analysis

The data are presented as mean ± standard deviation or median (lower quartile; upper quartile) as appropriate. The type of distribution was tested with the Shapiro-Wilk W test. Comparative analysis of two independent groups was performed with Mann-Whitney U test for quantitative data and Fisher's exact test for qualitative data. Comparative analysis of two dependent groups was performed with Wilcoxon signed-rank test. Spearman's rank correlation coefficient was used to assess correlation between the inflammatory biomarkers. Probability was considered significant at *p* < 0.05. All statistical tests were 2-tailed. Statistical analysis was performed with STATISTICA v. 7.0 (StatSoft Inc., USA) and IBM SPSS Statistics v. 26.0 (SPSS Inc., USA).

### Ethical approval

The study followed the Good Clinical Practice (GCP) standards and the principles of the Declaration of Helsinki. The study was approved by the ethics committee of the National Medical Research Centre of Cardiology named after academician E.I. Chazov of the Ministry of Health of the Russian Federation, Moscow, Russia. Written informed consent was obtained from all the participants.

## Results

### The assay measuring the mCRP level in blood plasma

**Figure 1:**
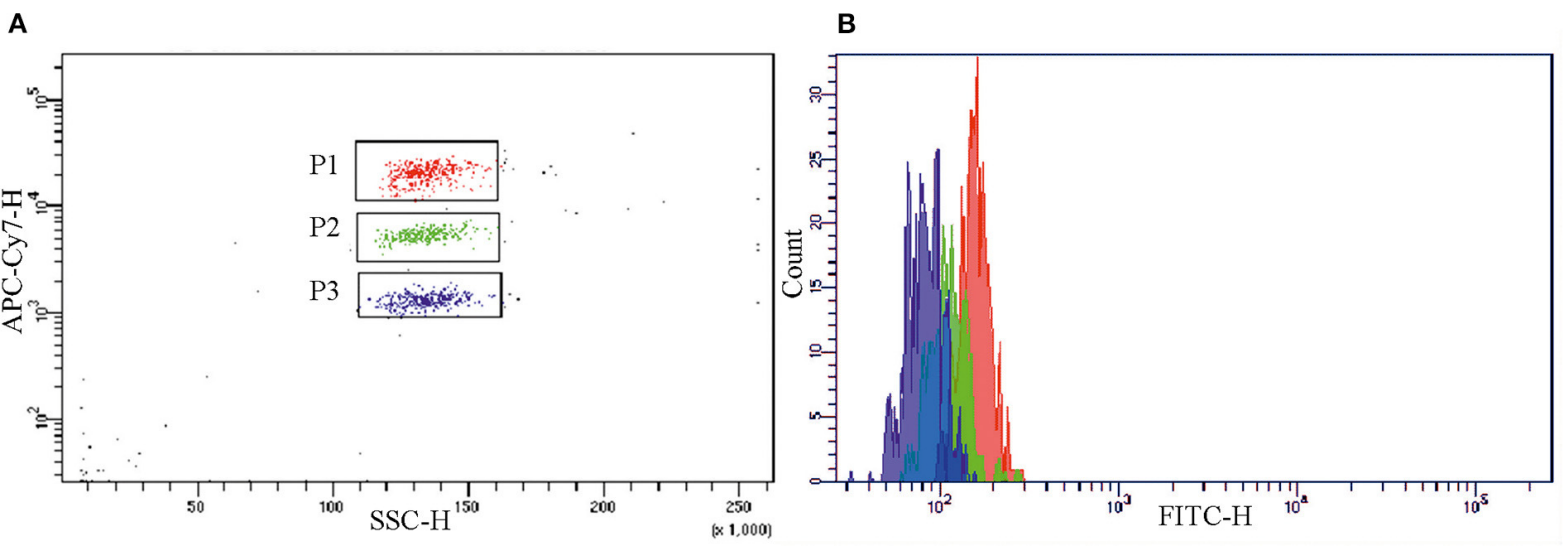
Background fluorescence intensity from the A5 (gate P1), C4 (gate P2) and E5 (gate P3) beads, conjugated to the monoclonal antibodies clones MOH 328 (anti-pCRP/mCRP), 8C8 (anti-mCRP), MOH372 (anti-pCRP), respectively, in **(A)** the APC-Cy7 channel and **(B)** the FITC channel in the absence of mCRP or pCRP and in the presence of polyclonal FITC-labeled antibody to CRP (GAHCRP-FITC). mCRP, monomeric C-reactive protein; pCRP, pentameric C-reactive protein.

The assay measuring the mCRP level in blood plasma was developed for flow cytometry analysis. The assay utilized the CBA beads C4 conjugated to the anti-mCRP mAb clone 8C8. The beads A5, conjugated to the anti-pCRP/mCRP mAb clone MOH328, and E5, conjugated to the anti-pCRP mAb clone MOH372, were used to rule out cross-reactivity of the mAb clone 8C8 to pCRP. To detect pCRP or mCRP the beads-antibody conjugates were incubated with samples of pCRP or mCRP solution in concentration 0.25 mg/L at room temperature for 1 h. Figure 1A shows a diagram of the background fluorescence intensity from the beads A5, C4, and E5 in the APC-Cy7 channel. Figure 1B shows a histogram of the fluorescence intensity from the beads A5, C4, and E5 in the FITC channel in the presence of the second-layer polyclonal anti-CRP antibody GAHCRP-FITC. Without any form of CRP, the mean fluorescence intensity (MFI) registered in the samples was low: 157 arbitrary units (a.u.), 112 a.u. and 82 a.u. for the A5, C4, and E5 beads, respectively.

**Figure 2:**
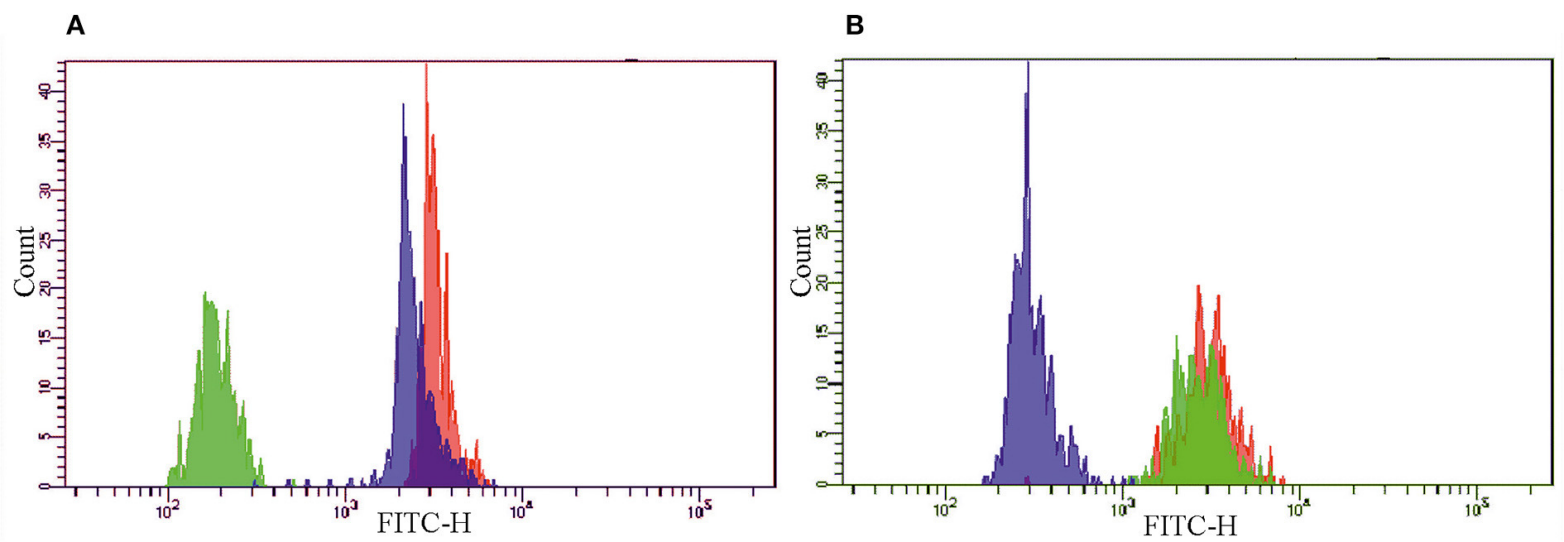
The histograms of the fluorescence intensity of the A5 (red), C4 (green) and E5 (blue) beads, conjugated to monoclonal antibodies clones MOH 328 (anti-pCRP/mCRP), 8C8 (anti-mCRP), MOH372 (anti-pCRP), respectively, in the presence of **(A)** pCRP or **(B)** mCRP in concentration 0.25 mg/L and polyclonal FITC-labeled antibody to CRP (GAHCRP-FITC). mCRP, monomeric C-reactive protein; pCRP, pentameric C-reactive protein.

Figure 2 shows MFI from the A5, C4, and E5 beads in the presence of pCRP (Figure 2A) or mCRP (Figure 2B) in concentration 0.25 mg/L and the second-layer polyclonal anti-CRP antibody GAHCRP-FITC. The A5 beads (gate P1), conjugated to the mAb clone MOH 328, bound pCRP and mCRP equally well. The C4 beads (gate P2), conjugated to the mAb clone 8C8, predominantly bound mCRP. The E5 beads (gate P3), conjugated to the mAb clone MOH372, predominantly bound pCRP. MFI recorded in the pCRP or mCRP samples was, respectively, 3,261 a.u. and 3,127 a.u. from the A5 beads, 188 a.u. and 2,766 a.u. from the C4 beads, 2,486 a.u. and 313 a.u. from the E5 beads in the FITC channel. Additionally, the mAb clone 8C8 was tested in a sample with pCRP concentration 5.0 mg/L. MFI from the C4 beads was 204 a.u. in the FITC channel in the presence of the second-layer polyclonal anti-CRP antibody GAHCRP-FITC. After mCRP was added into the sample up to concentration 0.25 mg/L, MFI raised to 2,842 a.u. Therefore, the mAb clone 8C8, conjugated to the C4 beads, is highly specific for mCRP.

The serial dilutions method was used to construct a calibration curve. The stock solution of the recombinant mCRP with known concentration in μg/L was serially diluted and titrated from 0.25 to 250.0 μg/L. Each diluted sample was incubated with the C4 beads and the second-layer polyclonal anti-CRP antibody GAHCRP-FITC. Then, MFI in the FITC channel from consecutively diluted samples was measured by flow cytometry. The relationship between MFI and the mCRP concentrations was linear at the mCRP concentrations from 1.0 to 100.0 μg/L. MFI did not differ significantly from the background fluorescence at the mCRP concentrations below 1.0 μg/L. Therefore, the mCRP concentration 1.0 μg/L shall be taken as a threshold value of the developed assay. The relationship between MFI and the mCRP concentrations above 100.0 μg/L was non-linear. Hence, to get a reliable result in a sample with the mCRP concentration more than 100.0 μg/L, this sample must be down-titrated until the mCRP concentration is within the range from 1.0 to 100.0 μg/L. From this concentration the actual mCRP level can be calculated considering the sample dilution factor.

### The association between the level of biomarkers (mCRP, hsCRP, IL-6, VWF) and the markers of subclinical carotid atherosclerosis

At the 7th-year follow-up survey plaque number increased in 45 (56%) patients (30 men and 15 women). Plaque number did not change in 35 (44%) patients (17 men and 18 women). The baseline characteristics of the patients are shown in Table 1.

**Table 1:** Baseline characteristics of the patients.

	**Patients with increased plaque number** **(*****n*** **=** **45)**	**Patients without increased plaque number** **(*****n*** **=** **35)**	***p***
Age, years	53 ± 6	53 ± 6	0.7
Men/women, no. (%)	30 (67%)/15 (33%)	17 (49%)/18 (51%)	0.1
Family history of premature CVD, no. (%)	9 (20%)	8 (23%)	0.8
Current smoking, no. (%)	12 (27%)	10 (29%)	0.5
Ex-smoker, no. (%)	6 (13%)	3 (9%)	0.9
BMI, kg/m^2^	26.0 (24.1; 30.7)	25.7 (24.3; 28.6)	0.7
BMI ≥ 30 kg/m^2^	14 (31%)	8 (23%)	0.4
TC, mmol/L	5.68 (5.12; 6.27)	5.70 (5.07; 6.25)	0.7
LDL-C, mmol/L	3.70 (3.20; 4.21)	3.80 (3.43; 4.34)	0.4
HDL-C, mmol/L	1.12 (1.0; 1.39)	1.10 (0.98; 1.32)	0.9
TG, mmol/L	1.54 (1.01; 2.10)	1.28 (1.0; 1.89)	0.5

*CVD, cardiovascular disease; BMI, body mass index; TC, total cholesterol; LDL-C, low-density lipoprotein cholesterol; HDL-C, high-density lipoprotein cholesterol; TG, triglycerides. Statistical analysis was performed with Mann-Whitney U test for quantitative data and Fisher's exact test for qualitative data*.

The groups did not differ in sex, current smokers, ex-smokers and non-smokers, family history of cardiovascular diseases (CVD), body mass index (BMI) and obesity. The patients did not have overt arterial hypertension, diabetes mellitus, nor they received cardiovascular medications at baseline. The patients with increased plaque number received atorvastatin in the dose 20 (20; 40) mg daily; the patients without increased plaque number received atorvastatin in the dose 20 (20; 40) mg daily (*p* = 0.7). The characteristics of the patients at the 7th-year follow-up survey are shown in Table 2.

**Table 2:** The characteristics of the patients at the 7th-year follow-up survey.

	**Patients with increased plaque number** **(*****n*** **=** **45)**	**Patients without increased plaque number** **(*****n*** **=** **35)**	***p***
Age, years	60 ± 6	60 ± 6	0.7
Men/women, no. (%)	30 (67%)/15 (33%)	17 (49%)/18 (51%)	0.1
Family history of premature CVD, no. (%)	9 (20%)	8 (23%)	0.8
Current smoking, no. (%)	11 (24%)	6 (17%)	0.4
Ex-smoker, no. (%)	7 (16%)	7 (20%)	0.9
BMI, kg/m^2^	27.1 (24.4; 31.4)	26.9 (25.2; 29.4)	0.7
BMI ≥ 30 kg/m^2^	16 (36%)	8 (23%)	0.2
Arterial Hypertension, no. (%)	22 (51%)	16 (43%)	0.8
Diabetes mellitus type 2, no. (%)	6 (14%)	1 (3%)	0.1
MACE, total no. (%)	5 (11%)	2 (6%)	0.4
- Myocardial Infarction, no. (%)	2 (4%)	0	
- Angina Pectoris, no. (%)	2 (4%)	2 (6%)	
- Stroke, no. (%)	1 (2%)	0	
TC, mmol/L	4.14 (3.84; 4.35)	3.99 (3.63; 4.38)	0.2
LDL-C, mmol/L	2.33 (2.05; 2.44)	2.15 (1.82; 2.34)	0.04
HDL-C, mmol/L	1.24 (1.08; 1.46)	1.22 (1.02; 1.48)	0.4
TG, mmol/L	1.22 (0.95; 1.61)	1.11 (0.88; 1.35)	0.6
mCRP, μg/L	6.3 (4.2; 9.8)	4.0 (2.45; 5.35)	0.0006
hsCRP, mg/L	1.2 (0.7; 2.4)	0.9 (0.6; 1.6)	0.2
hsCRP ≥ 2.0 mg/L	18	8	0.1
IL-6, pg/mL	0.0 (0.0; 3.2)	0.0 (0.0; 2.14)	0.6
VWF, IU/dL	107 (77; 163)	103 (85; 124)	0.6

*CVD, cardiovascular disease; BMI, body mass index; MACE, major adverse cardiovascular events; TC, total cholesterol; LDL-C, low-density lipoprotein cholesterol; HDL-C, high-density lipoprotein cholesterol; TG, triglycerides; mCRP, monomeric C-reactive protein; hsCRP, C-reactive protein measured with high-sensitivity test; IL-6, interleukin-6; VWF, von Willebrand factor. Statistical analysis was performed with Mann-Whitney U test for quantitative data and Fisher's exact test for qualitative data*.

In the patients with increased plaque number MACE developed in 5 cases (2 cases of myocardial infarction, 2 cases of angina pectoris and 1 case of stroke) during the follow-up period. Moreover, diabetes mellitus type 2 developed in 6 cases and arterial hypertension requiring pharmacological treatment in 22 cases. In the patients without increased plaque number MACE developed in 2 cases (angina pectoris in both cases) during the follow-up period. Also, diabetes mellitus type 2 developed in 1 case and arterial hypertension requiring pharmacological treatment in 16 cases.

**Figure 3:**
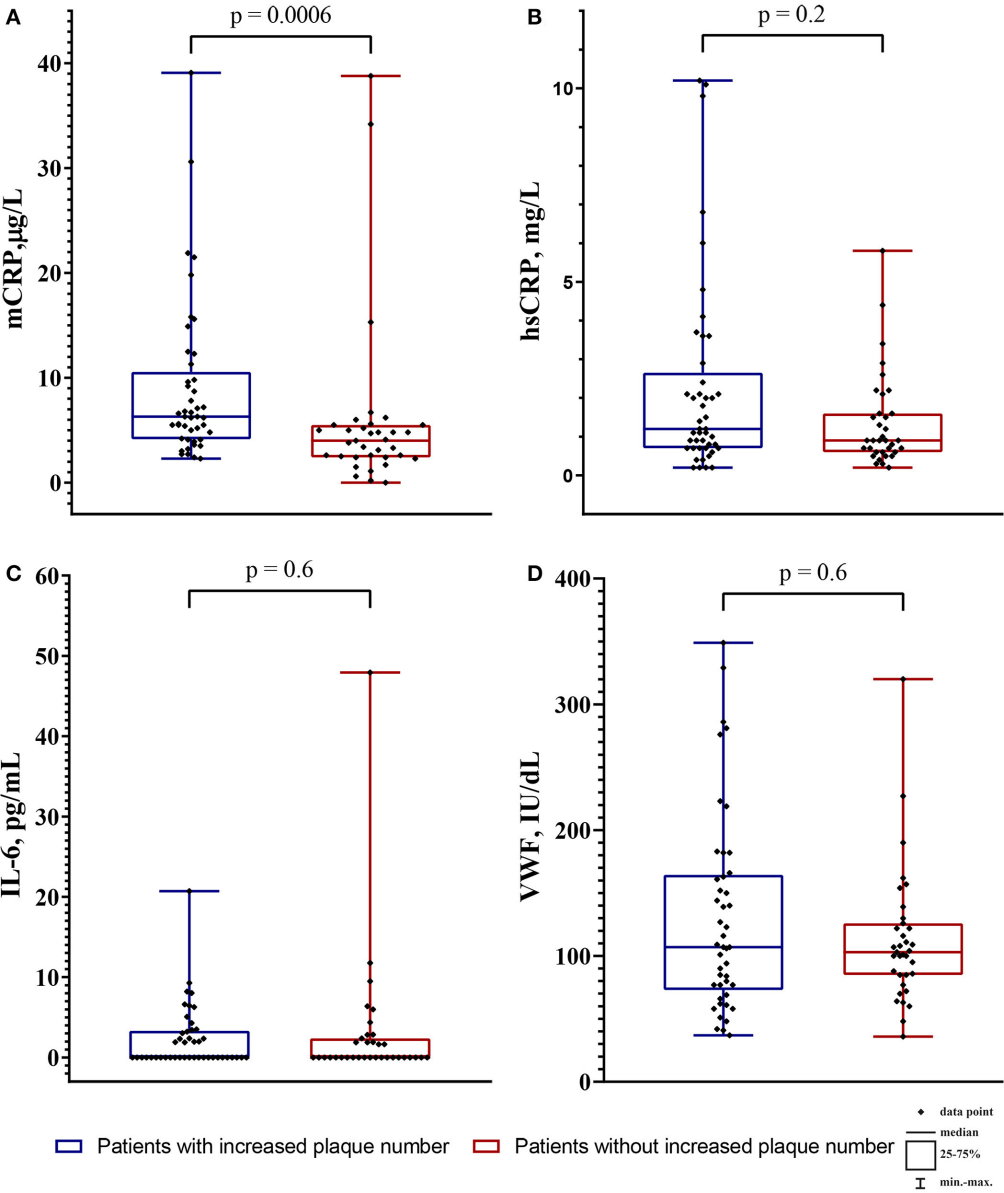
The level of biomarkers in the patients with increased plaque number (blue boxplots) and the patients without increased plaque number (red boxplots). **(A)** the monomeric C-reactive protein (mCRP) level in μg/L; **(B)** the high-sensitivity C-reactive protein (hsCRP) level in mg/L; **(C)** the interleukin-6 level (IL-6) in pg/mL; **(D)** the von Willebrand factor (VWF) level in IU/dL. The intergroup difference was analyzed by Mann-Whitney U test.

The groups were different only in the LDL-C level and the mCRP level. The level of biomarkers depending on the increase in plaque number is shown in Figure 3.

In all patients (*n* = 80) the level of mCRP was 5.2 (3.3; 7.1) μg/L, hsCRP 1.05 (0.7; 2.1) mg/L, IL-6 0.0 (0.0; 2.8) pg/mL, VWF 1.06 (0.77; 1.51) IU/dL. The mCRP level did not correlate with the hsCRP level (*r* = 0.006, *p* = 0.9) and the IL-6 level (*r* = 0.02; *p* = 0.9), but positively correlated with the VWF level (*r* = 0.3; *p* = 0.01). The hsCRP level positively correlated with the IL-6 level (*r* = 0.4; *p* = 0.001). The correlation matrix between mCRP and other biomarkers are shown in Table 3.

**Table 3:** The correlation matrix between mCRP and other biomarkers.

**Variables**	**mCRP**		**hsCRP**		**IL-6**		**VWF**	
	***r***	***p***	***r***	***p***	***r***	***p***	***r***	***p***
mCRP	1	–	0.006	0.9	0.02	0.9	0.3	0.01
hsCRP	0.006	0.9	1	–	0.4	0.001	0.1	0.2
IL-6	0.02	0.9	0.4	0.001	1	–	−0.1	0.2
VWF	0.3	0.01	0.1	0.2	−0.1	0.2	1	–

*mCRP, monomeric C-reactive protein; hsCRP, C-reactive protein measured with high-sensitivity test; IL-6, interleukin-6; VWF, von Willebrand factor. Spearman's rank correlation coefficient was used to measure r and p-values. The bold values mean the values with p <0.05*.

The patients were divided by the median mCRP level 5.2 (3.3; 7.1) μg/L. The group with the mCRP level <5.2 μg/L comprised 39 patients (21 men and 18 women). The group with the mCRP level ≥ 5.2 μg/L comprised 41 patients (26 men and 15 women). The increase in plaque number was detected in 14 (36%) patients in the group with the mCRP level <5.2 μg/L and in 31 (76%) patients in the group with the mCRP level ≥ 5.2 μg/L. The ultrasound markers of carotid atherosclerosis and CCA-IMT depending on the mCRP level are shown in Table 4.

**Table 4:** The ultrasound markers of carotid atherosclerosis and CCA-IMT depending on the mCRP level.

	**mCRP** **<** **5.2** **μg/L (*****n*** **=** **39)**			**mCRP** **≥5.2** **μg/L (*****n*** **=** **41)**		
	**Baseline**	**The 7th-year follow-up survey**	**Change**	**Baseline**	**The 7th-year follow-up survey**	**Change**
Plaque number	3.0 (1.0; 4.0)	3.0 (2.0; 4.0)	0.0 (0.0; 1.0)*	2.0 (1.0; 3.0)	3.0 (2.0; 4.0)	1.0 (1.0; 2.0)*
Total plaque height, mm	5.56 (2.57; 8.13)	6.21 (3.97; 10.13)	0.22 (−0.24; 1.91)*	3.70 (2.34; 5.51)	7.04 (4.45; 11.30)	1.97 (1.14; 3.14)*
CCA-IMT right, mm	0.66 (0.58; 0.71)	0.70 (0.61; 0.79)	0.04 (0.01; 0.09)*	0.72 (0.62; 0.81)	0.77 (0.65; 0.89)	0.06 (−0.01; 0.13)*
CCA-IMT left, mm	0.62 (0.60; 0.75)	0.70 (0.61; 0.84)	0.04 (0.01; 0.09)*	0.67 (0.61; 0.78)	0.72 (0.61; 0.91)	0.03 (-0.04; 0.07)^NS^
CCA-IMT mean, mm	0.66 (0.58; 0.72)	0.70 (0.63; 0.82)	0.05 (0.0; 0.1)*	0.71 (0.64; 0.80)	0.74 (0.65; 0.87)	0.05 (0.00; 0.1)*

mCRP, monomeric C-reactive protein; CCA-IMT, intima-media thickness of common carotid artery; *p <0.05; ^NS^, not significant (Wilcoxon signed-rank test).

**Figure 4:**
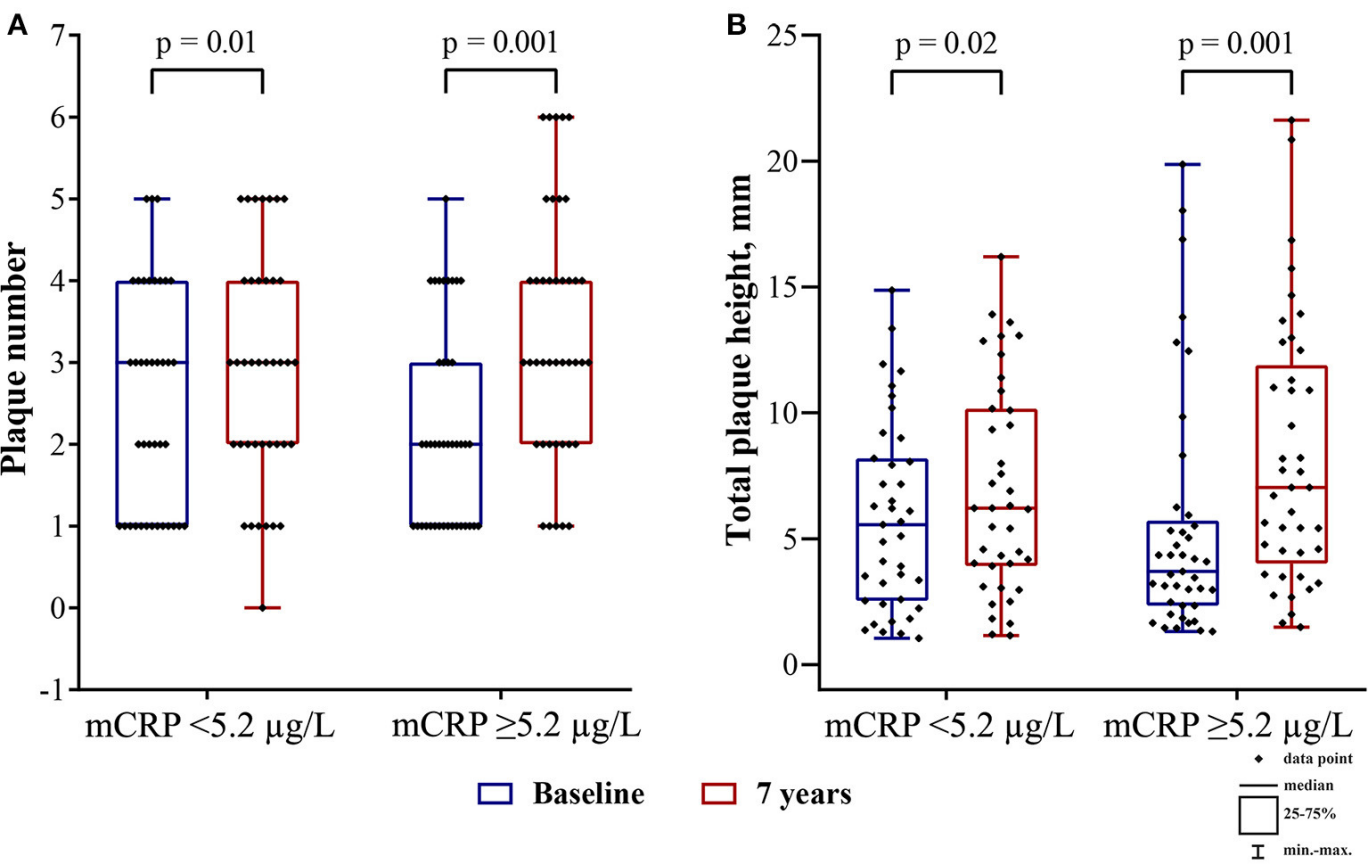
**(A)** plaque number and **(B)** total plaque height at baseline (blue boxplots) and the 7th-year follow-up survey (red boxplots) in the patients with the mCRP level below median or the median mCRP level or higher. At the 7th-year follow-up survey, the intragroup change in all markers was significant (Wilcoxon signed-rank test). mCRP, monomeric C-reactive protein.

The association between the mCRP level and the change in plaque number, total plaque height (Figure 4), left, right and mean CCA-IMT was significant for the patients with the mCRP level <5.2 μg/L, as well as the patients with the mCRP level ≥ 5.2 μg/L, except for left CCA-IMT, which did not change significantly in the patients with the mCRP level ≥ 5.2 μg/L. However, the patients with the mCRP level ≥ 5.2 μg/L demonstrated a pronounced increase in the ultrasound markers of carotid atherosclerosis. In the patients with the mCRP level <5.2 μg/L, the median percentage change in plaque number was 0%, total plaque height 4%, right CCA-IMT 7%, left CCA-IMT 5%, mean CCA-IMT 5%, whereas in the patients with the mCRP level ≥ 5.2 μg/L the median percentage change in plaque number was 50%, total plaque height 57%, right CCA-IMT 7%, mean CCA-IMT 7%.

The unadjusted odds ratio for the formation of new carotid atherosclerotic plaques was 5.5 [95% confidence interval (CI) 2.1; 14.6; *p* = 0.001] for the patients with the median mCRP level or higher. Logistic regression analysis was performed to adjust the odds ratio for other risk factors and biomarkers. Regression analysis was performed by stepwise inclusion or exclusion of factors in the model. The resulting logistic regression model included the mCRP level ≥ 5.2 μg/L, the hsCRP level, the LDL-C level at the 7th-year follow-up survey and male sex. Other risk factors and biomarkers were excluded, because they decreased the predictive value of the model. The model was significant (*p* = 0.001) with 75.0% of correct predictions for the formation of new carotid atherosclerotic plaques. The model with the hsCRP level ≥ 2.0 mg/L as a categorical variable instead of the hsCRP level as a continuous variable was also significant (*p* = 0.001), but returned slightly less correct predictions (71.3%). The adjusted odds ratio for the formation of new carotid atherosclerotic plaques was 4.74 (95% CI 1.70–13.24) for the patients with the median mCRP level or higher. The results of the logistic regression model predicting the formation of new atherosclerotic plaques are shown in the Table 5.

**Table 5:** The results of the logistic regression model predicting the formation of new atherosclerotic plaques.

**Predictor**	**Coefficient (β)**	**aOR (95% CI)**	***p***
Intercept	−4.00	0.02	0.02
The mCRP level ≥ 5.2 μg/L	1.56	4.74 (1.70–13.24)	0.001
The hsCRP level, mg/L	0.30	1.35 (0.98–1.86)	0.06
The achieved LDL-C level, mmol/L	1.14	3.12 (0.69–14.08)	0.14
Male sex	0.94	2.57 (0.90–7.36)	0.08

*aOR, adjusted odds ratio; CI, confidence interval; mCRP, monomeric C-reactive protein; hsCRP, C-reactive protein measured with high-sensitivity test; LDL-C, low-density lipoprotein cholesterol*.

## Discussion

The assay measuring the mCRP level in blood plasma was developed in this study. The assay utilized the CBA beads conjugated to the mAb clone 8C8. Test measurements performed in samples of pCRP and the recombinant mCRP in the range of concentrations confirmed high specificity of the mAb clone 8C8 for mCRP. The calibration curve for the developed assay was constructed with the serial dilutions method in consecutively diluted samples of the recombinant mCRP. The relationship between MFI and the mCRP concentrations was linear in the range of the mCRP concentrations from 1.0 μg/L to 100.0 μg/L. At the mCRP concentrations below 1.0 μg/L, MFI did not differ from the background fluorescence. Therefore, the threshold sensitivity of the developed assay is 1.0 μg/L. The high specificity of the mAb clone 8C8 to mCRP was previously shown in a number of studies (15, 18, 19, 21, 22). The threshold sensitivity of assays measuring the mCRP level also was 1.0 μg/L for ELISA utilizing mAb clone 8C8 in the study of Zhang et al. (18) and for ELISA utilizing mAb produced by Wang et al. (17).

The presence of carotid atherosclerosis in asymptomatic individuals with low to moderate cardiovascular SCORE risk may reclassify them to a higher risk category. Such individuals may need a more aggressive approach to the modification of risk factors, including initiation of lipid-lowering therapy, to prevent or halt MACE development (4, 23). All the patients enrolled in the study had subclinical low-grade carotid stenoses, detected with ultrasonography, narrowing the arterial lumen <50%, and the LDL-C level higher than 2.6 mmol/L. The risk category was reclassified to high cardiovascular SCORE risk according to ESC/EAS Guidelines for the Management of Dyslipidaemias that were applicable at the time of enrollment (23). The target LDL-C level in this risk category was <2.6 mmol/L. All the patients enrolled in the study were prescribed atorvastatin and achieved the target LDL-C level. Since the patients had no evidence of advanced carotid stenoses, assessment of atherosclerosis progression by change in blood flow parameters was inapplicable. Therefore, the increase in plaque number was considered as the main ultrasound marker of carotid atherosclerosis progression in this study (27).

Despite lipid-lowering therapy, carotid plaque number increased in 45 (56%) patients by the 7th-year follow-up survey. The patients with and without increased plaque number were different only in the LDL-C level and the mCRP level. The median mCRP level 5.2 μg/L or higher corresponded with the pronounced increase in plaque number and total plaque height. The adjusted odds ratio for the formation of new carotid atherosclerotic plaques was 4.74 (95% CI 1.70–13.24) for the patients with the median mCRP level or higher. Higher plaque number was associated with increased stroke (28) and CAD (29) occurrence in asymptomatic individuals. An increase in maximum plaque thickness was associated with increased CAD (30) occurrence in asymptomatic individuals. The maximum plaque thickness was equal to the plaque burden in prediction of MACE in asymptomatic individuals (31). An association between the increase in CCA-IMT and higher cardiovascular risk in asymptomatic individuals was shown in large population studies (32, 33).

Correlation analysis showed that the mCRP level did not correlate with the hsCRP and IL-6 level, yet weakly correlated with the VWF level. Williams et al. also reported that the mCRP level did not correlate with the hsCRP level (19). Zhang et al. reported that in patients with autoimmune skin diseases and the elevated mCRP level, the hsCRP level was within the normal range (18). Munuswamy et al. also did not find correlation of the mCRP level with the hsCRP level in patients with COPD (20).

In this study population, the median hsCRP level was 1.05 (0.7; 2.1) mg/L, which was well below the 2.0 mg/L threshold for residual inflammatory cardiovascular risk (4). Of 26 patients who had the hsCRP level ≥ 2.0 mg/L, 18 belonged to the group with increased plaque number and 8 to the group without change in plaque number. The median IL-6 level was 0.0 (0.0; 2.8) pg/mL. The median VWF level was 106 (77; 151) IU/dL, which was within normal limits.

To our knowledge, this is the first study evaluating the association of the mCRP level with ultrasound markers of carotid atherosclerosis. Previously, Wang et al. measured the mCRP level in blood plasma of patients with acute myocardial infarction, unstable and stable angina pectoris (17). The highest mCRP level was observed in patients with acute myocardial infarction (20.96 ± 1.64 μg/L), especially in those who died in 30 days after the event (36.70 ± 10.26 μg/L), whereas in patients with stable angina pectoris or healthy individuals mCRP was not detected (17). Zhang et al. reported the median mCRP level in blood plasma of patients with eczema, psoriasis, urticaria and healthy controls ranging from 15.2 to 59.8 μg/L (18). Williams et al. detected the mean mCRP level 1,030.0 ± 110.0 μg/L in serum of patients with acute inflammation and the hsCRP level more than 100 mg/L (19). Munuswamy et al. reported that the median mCRP level in patients with COPD was 660.0 μg/L, whereas in healthy controls mCRP was not traceable (20). In our study, the mCRP level was lower: 6.3 (4.2; 9.8) μg/L vs. 4.0 (2.45; 5.35) μg/L in the patients with and without increased plaque number, respectively. This may be attributed to the absence of comorbidity, few traditional cardiovascular risk factors and statin treatment in the patients.

The association of the elevated hsCRP level with an increase in the degree of carotid artery stenoses, greater number, larger area and total height of carotid atherosclerotic plaques was shown in a number of studies (27, 34–36). The association of the hsCRP level with cardiovascular risk was shown in a number of large randomized controlled clinical trials, including JUPITER, CANTOS, PROVE-IT, and IMPROVE-IT (3). The association of the elevated IL-6 level with a greater plaque number (37) and a greater CCA-IMT (38) was reported in asymptomatic individuals. The IL-6 level was associated with the cardiovascular risk in the CANTOS trial (3). The elevated VWF level was associated with the endothelial disfunction (39, 40) and higher cardiovascular risk in individuals with subclinical carotid atherosclerosis (41). As it was already mentioned, the overall health status of the patients in our study was considerably mild, which may explain normal level of the inflammatory biomarkers and VWF. Moreover, the patients were prescribed statins, which reduce the hsCRP level (3). Based on the hsCRP level, the participants of this study should have been classified to the low residual inflammatory cardiovascular risk category. Yet, the mCRP level allowed to identify individuals with higher probability of formation of new carotid atherosclerotic plaques among the study participants.

## Limitations of the study

The main limitation of this study is the absence of baseline measurement of the biomarkers and low rate of MACE. This precludes from correct assessment of the mCRP level as a biomarker of residual inflammatory risk.

## Conclusion

Despite statin treatment, 56% of the patients with low-grade carotid stenoses and initially moderate cardiovascular SCORE risk demonstrated the increase in carotid plaque number. The patients with and without increased plaque number were different only in the LDL-C level and the mCRP level. The level of hsCRP, IL-6 and VWF was normal. The median mCRP level or higher allowed to identify individuals with higher probability of formation of new carotid atherosclerotic plaques among the study participants. The adjusted odds ratio for the formation of new carotid atherosclerotic plaques was 4.74 (95% CI 1.70–13.24) for the patients with the median mCRP level or higher. Therefore, this study for the first time shows that the higher mCRP level is associated with the more pronounced increase in plaque number and total plaque height in patients with normal level of traditional inflammatory biomarkers and initially moderate SCORE risk.

## Data availability statement

The raw data supporting the conclusions of this article will be made available by the authors, without undue reservation.

## Ethics statement

The studies involving human participants were reviewed and approved by the Ethics Committee of the National Medical Research Centre of Cardiology named after academician E.I. Chazov of the Ministry of Health of the Russian Federation. The patients/participants provided their written informed consent to participate in this study.

## Author contributions

IM, SK, TB, and ZG: conceptualization. OP, LK, TB, YA, MZ, and EM: data curation. EM and ZG: formal analysis. IM, LP, and ZG: funding acquisition. IM, OP, MT, LK, OS, YA, MZ, and TK: investigation. SK, TB, OS, TK, and ZG: methodology. MT and ZG: project administration. LK and LP: resources. SK, TB, and ZG: supervision. OS, EM, and LP: validation. IM, MT, and OS: visualization. IM, YA, MZ, and TK: writing—original draft. IM, SK, OP, and ZG: writing—review and editing. All authors have read and agreed to the published version of the manuscript.

## Funding

This research was funded by the Russian Science Foundation (RSF) project #21-15-00029.

## Conflict of interest

The authors declare that the research was conducted in the absence of any commercial or financial relationships that could be construed as a potential conflict of interest.

## Publisher's note

All claims expressed in this article are solely those of the authors and do not necessarily represent those of their affiliated organizations, or those of the publisher, the editors and the reviewers. Any product that may be evaluated in this article, or claim that may be made by its manufacturer, is not guaranteed or endorsed by the publisher.
